# NSUN5 and RNA m^5^C epitranscriptomic regulation in tumor progression

**DOI:** 10.3389/fcell.2026.1771110

**Published:** 2026-01-30

**Authors:** Qiao Zhou, Chunhong Li, Xiulin Jiang, Yixiao Yuan, Qiang Zhou, Qiang Wang, Lili Jiang

**Affiliations:** 1 Department of Urology, Aerospace Center Hospital, Beijing, China; 2 Department of Oncology, Suining Central Hospital, Suining, Sichuan, China; 3 Department of Systems Biology, City of Hope Comprehensive Cancer Center, Monrovia, CA, United States; 4 Department of Gastrointestinal Surgical Unit, Suining Central Hospital, Suining, Sichuan, China

**Keywords:** biomarker, chemoresistance, epitranscriptomic crosstalk, metabolic reprogramming, NSUN5, RNA m5C methylation, therapeutic target, tumor microenvironment

## Abstract

NSUN5 is a pivotal RNA 5-methylcytosine (m^5^C) methyltransferase that predominantly catalyzes site-specific m^5^C modifications in ribosomal RNA (rRNA), thereby regulating ribosome assembly, selective translation, and cellular stress adaptation. Recent studies have demonstrated that NSUN5 is aberrantly expressed in multiple cancer types, and its upregulation is often associated with advanced tumor stage, poor prognosis, and immune evasion. Mechanistically, NSUN5 modulates the m^5^C modification of rRNA or specific mRNAs, reshaping the cellular proteome and influencing tumor cell proliferation, migration, invasion, and stemness maintenance. Moreover, NSUN5 participates in tumor metabolic reprogramming, including glycolysis and lipid biosynthesis, as well as in cellular stress responses and resistance to chemotherapy and radiotherapy. NSUN5 can also mediate tumor microenvironmental regulation through RNA modifications, such as modulating macrophage polarization, enhancing antioxidative capacity, and facilitating immune escape. Functional crosstalk exists between NSUN5 and other RNA epigenetic regulators, forming a complex regulatory network. Given its central role and structural features, NSUN5 represents a potential therapeutic target and biomarker. Direct strategies include small-molecule inhibitors that block its methyltransferase activity, whereas indirect approaches focus on interfering with downstream signaling pathways or synergizing with other RNA modifications to inhibit tumor progression. Additionally, NSUN5 expression may serve as a stratification marker for patient classification and treatment response prediction, supporting precision oncology. Future research should focus on genome-wide target identification, integration of single-cell and spatial transcriptomics, and mechanism-driven drug development to advance the clinical translation of NSUN5-targeted interventions.

## 1 Introduction

With the rapid advancement of high-throughput sequencing technologies and chemical labeling approaches, RNA epitranscriptomics has emerged as a frontier in cancer biology research (Jiang et al., 2021). Unlike DNA and histone modifications, RNA modifications can dynamically regulate RNA splicing, stability, localization, and translation efficiency without altering the nucleotide sequence, thereby fine-tuning gene expression programs (Yuan et al., 2025). Accumulating evidence indicates that aberrant RNA modifications are prevalent across diverse tumor types and play critical roles in tumor initiation, progression, metabolic reprogramming, and therapeutic resistance (Wang et al., 2025).

To date, more than 170 chemical RNA modifications have been identified, among which m^6^A, m^5^C, and pseudouridine (Ψ) have been most extensively studied (Chen et al., 2024). Compared with m^6^A, research on RNA m^5^C modification in cancer has started relatively late but has gained increasing attention in recent years (Chen et al., 2025). m^5^C was first identified in tRNA and rRNA and was later confirmed to be widespread in mRNAs and various non-coding RNAs, participating in crucial biological processes such as RNA stability, nuclear export, and translation regulation (Li et al., 2025). The dynamic regulation of m^5^C depends on a sophisticated network of enzymes, mainly including “writers,” “readers,” and potentially “erasers (Li et al., 2025).” Known m^5^C writers predominantly consist of NSUN family proteins and DNMT2, both of which mediate site-specific m^5^C formation in an S-adenosylmethionine-dependent manner (Feng et al., 2023). Correspondingly, m^5^C readers specifically recognize this modification and mediate downstream functional outputs, whereas the existence of classical m^5^C erasers remains controversial, suggesting that m^5^C modifications are relatively stable yet finely regulated. Current studies indicate that the RNA m^5^C reader proteins mainly include YBX1, YBX2, ALYREF, and SRSF2, which specifically recognize m^5^C-modified RNAs and participate in the regulation of mRNA stability, nuclear export, and alternative splicing, thereby governing key post-transcriptional processes (Ma et al., 2023; Zhang et al., 2024a).

**FIGURE 1:**
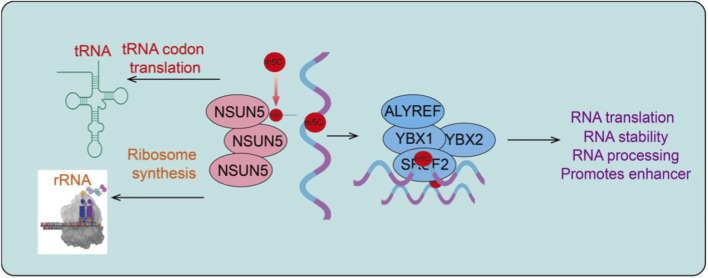
Molecular mechanisms of NSUN5-mediated RNA m5C modification. Diagram depicting the molecular functions of NSUN5 in RNA metabolism. NSUN5 catalyzes m^5^C modification on multiple RNA species, including tRNA, rRNA, and mRNA. m^5^C modification of tRNA regulates codon–anticodon interactions and translation efficiency, while rRNA methylation influences ribosome synthesis and function. NSUN5-mediated m^5^C marks on mRNA are recognized by m^5^C reader proteins such as ALYREF, YBX1, YBX2, and SRSF2, leading to altered RNA stability, processing, nuclear export, and translation. Through these coordinated mechanisms, NSUN5-mediated RNA m^5^C modification fine-tunes gene expression programs that support tumor growth, adaptation, and malignancy.

Members of the NSUN family, including NSUN1–7, play central roles in RNA m^5^C modification, exhibiting distinct substrate preferences, subcellular localization, and biological functions (Li et al., 2022). Among them, NSUN5, a relatively understudied m^5^C methyltransferase, has recently emerged as a key regulator in cancer biology (Sun et al., 2020). Unlike other NSUN family members, which predominantly target tRNAs or mRNAs, NSUN5 mainly mediates rRNA m^5^C modification and, by influencing ribosome function and selective translation, contributes to cellular stress adaptation and tumor progression (Sun et al., 2020). Growing evidence suggests that aberrant NSUN5 expression is closely associated with the development and prognosis of multiple tumor types (Zhou et al., 2023). In this review, we systematically summarize current knowledge regarding NSUN5 in cancer, highlighting its functional roles and underlying molecular mechanisms in tumor progression. Furthermore, we discuss potential strategies for targeting NSUN5 and the prospects for clinical translation, aiming to provide new insights and theoretical foundations for RNA modification-mediated cancer therapy (Figure 1).

## 2 Molecular characteristics and biological functions of NSUN5

### 2.1 Gene and protein structure of NSUN5

NSUN5 is a member of the NSUN family, encoding an S-adenosylmethionine (SAM)-dependent RNA methyltransferase (Zhou et al., 2023). In the human genome, the NSUN5 gene is located in a specific chromosomal region, and its sequence is highly conserved across eukaryotes, with homologs detectable from yeast to mammals, suggesting that NSUN5 performs fundamental and evolutionarily conserved biological functions (Ding et al., 2022). This high degree of evolutionary conservation supports its critical role in maintaining ribosome function and cellular homeostasis. At the protein structural level, NSUN5 is a canonical RNA m^5^C methyltransferase, with a core domain that adopts a highly conserved Rossmann-like fold. This domain facilitates binding to the methyl donor SAM and catalyzes the methylation reaction, a feature shared by all NSUN family members and essential for their function as m^5^C “writers (Liu et al., 2022).” Compared with other NSUN proteins, NSUN5 exhibits a relatively compact structure and lacks prominent auxiliary RNA-binding domains, which likely underlies its substrate preference for rRNA. The catalytic activity of NSUN5 depends on several highly conserved amino acid residues, particularly a cysteine residue located in the catalytic center, which is indispensable for m^5^C formation (Xu et al., 2025). This cysteine forms a covalent intermediate with the cytosine in the RNA substrate, ensuring precise methyl transfer. In addition, NSUN5 contains conserved motifs involved in SAM binding and substrate positioning, collectively guaranteeing the specificity and efficiency of its methyltransferase activity (Chen et al., 2019). Notably, these structural features not only define NSUN5 enzymatic function but also provide a theoretical basis for drug targeting. The well-defined SAM-binding pocket and catalytic core render NSUN5 amenable to inhibition by small-molecule compounds, an aspect of practical significance for subsequent discussions on therapeutic strategies (Chen et al., 2019).

### 2.2 Characteristics of NSUN5-Mediated RNA m^5^C modification

As a key RNA m^5^C writer, NSUN5 exhibits distinct substrate specificity and site selectivity compared with other NSUN family members (Chen et al., 2019). Current evidence indicates that NSUN5 primarily targets ribosomal RNA (rRNA), especially highly conserved sites within the large subunit rRNA (Chen et al., 2019). These modification sites are evolutionarily conserved across species, underscoring the fundamental role of NSUN5-mediated rRNA m^5^C in ribosome structural stability and translational function (Chen et al., 2019).

Beyond rRNA, emerging evidence suggests that NSUN5 may also contribute to mRNA m^5^C modification. High-throughput sequencing data indicate that NSUN5 can methylate specific mRNAs under certain cellular conditions or stress states, potentially influencing mRNA stability, translation efficiency, or subcellular localization (Zha et al., 2024). Although the exact repertoire and distribution of NSUN5-modified mRNAs remain to be systematically validated, these observations provide a mechanistic basis for subsequent findings in which NSUN5-mediated mRNA modifications affect cellular responses and tumor-related translation programs. Compared with NSUN2, which primarily targets tRNA and mRNA, NSUN5 appears to regulate the global translation program indirectly through ribosome modification rather than directly controlling individual transcript fate. In terms of site specificity, NSUN5-catalyzed m^5^C modifications typically occur at structurally conserved cytosine residues located in functionally critical regions of rRNA, such as the ribosomal subunit assembly sites and peptidyl transfer center (Zha et al., 2024). This selective modification allows NSUN5 to fine-tune ribosome performance and indirectly influence the translational efficiency of specific mRNAs, thereby enabling “translation reprogramming.” Functionally, NSUN5 differs from other m^5^C writers: NSUN2 primarily acts on tRNA and mRNA, regulating RNA stability, nuclear export, and cell proliferation, while DNMT2, though possessing methyltransferase activity, has a limited substrate range, mainly modifying specific tRNAs (Zha et al., 2024). In contrast, NSUN5 preferentially impacts overall ribosome function through rRNA modification, providing a unique advantage in stress adaptation and tumor-associated translational regulation (Zha et al., 2024).

### 2.3 Physiological functions of NSUN5

NSUN5 functions as a S-adenosylmethionine (SAM)-dependent RNA methyltransferase, catalyzing the site-specific methylation of cytosine residues in rRNA to form 5-methylcytosine (Heissenberger et al., 2019). This enzymatic activity is critical for ribosome biogenesis and functional fidelity, as NSUN5-mediated methylation occurs at structurally conserved sites within the large subunit rRNA that are essential for ribosomal assembly and the peptidyl transferase reaction (Jiang et al., 2020a). By stabilizing rRNA structure and optimizing ribosome conformation, NSUN5 indirectly regulates the efficiency and accuracy of global protein translation, which is particularly important under cellular stress conditions (Jiang et al., 2020a). Beyond ribosomal RNA, emerging evidence suggests that NSUN5 may also catalyze m^5^C modification on selected mRNAs, thereby modulating mRNA stability, translational efficiency, or subcellular localization in a context-dependent manner (Hu et al., 2025). Through these dual activities on rRNA and mRNA, NSUN5 contributes to cellular stress adaptation, proliferation control, and tumor-associated translational reprogramming, highlighting its central role in maintaining cellular homeostasis and regulating gene expression at the post-transcriptional level (Hu et al., 2025). Under physiological conditions, NSUN5 is expressed across multiple tissues and cell types, generally at stable levels, consistent with its role in fundamental cellular processes (Zhang et al., 2024b). Unlike certain NSUN family members that exhibit tissue-specific high expression, NSUN5 expression primarily maintains basal translational activity and cellular homeostasis rather than driving specialized differentiation programs. This ribosome-centric regulatory mode also provides a foundation for understanding the pathological roles of NSUN5, particularly in tumor progression.

## 3 Molecular mechanisms of NSUN5 in cancer progression

In Table 1, we summarizes the multifaceted roles of NSUN5 in cancer biology, highlighting its involvement in tumor metabolism, stress adaptation, proliferation, and progression.

**TABLE 1:** Multifaceted roles of NSUN5 in cancer biology.

Cancer type	NSUN5 function	Molecular mechanism/Axis	Biological outcome	Reference
ccRCC	Promotes glycolysis	NSUN5 → m^5^C modification of ENO3 mRNA → increased stability/protein	Enhanced glucose uptake, lactate production, proliferation	Zhang et al. (2023)
Pca	Lipid metabolic reprogramming	CDK13 → NSUN5 (Ser327) → stabilizes ACC1 mRNA → nuclear export	Fatty acid synthesis, tumor growth	Han et al. (2025a)
HCC	Tumor proliferation and glycolysis	NSUN5 → m^5^C modification of EFNA3 mRNA	Increased proliferation and glycolytic flux	Gu et al. (2024)
HCC	Cell proliferation	NSUN5 → m^5^C of ZBED3 mRNA → Wnt/β-catenin signaling activation	Enhanced tumor growth	Jiang et al. (2020b)
CRC	Cell cycle regulation and chemoresistance	NSUN5 → CDK4/CDK6/CCNE1 & BRCA2/BRIP1	Proliferation, chemoresistance	Shu et al. (2025)
GBM	Stemness and proliferation	NSUN5 → m^5^C of 28S rRNA	Increased protein synthesis, sphere formation, migration, TMZ resistance	Zhou et al. (2023)
CCA	Metabolism and migration	NSUN5 → m^5^C of GLS mRNA (137C)	Proliferation, migration, invasion, cuproptosis resistance	Han et al. (2025b), Chen et al. (2023)
HCC	EMT & metastasis	NSUN5 → recruits WDR5 → H3K4me3 at SMAD3 promoter	Enhanced migration and invasion	Li et al. (2023)
CRC	Immune regulation	NSUN5 → m^5^C of GPX4 mRNA → cGAS-STING activation	Enhanced anti-tumor immune response	Liu et al. (2024)
Glioma	Immune regulation	NSUN5 → m^5^C of CTNNB1 caRNA → TET2 oxidation → RBFOX2-mediated degradation	Suppressed β-catenin, enhanced TAM phagocytosis	Janin et al. (2019)
GC	Proliferation, stemness, immune evasion	NSUN5 → Wnt/β-catenin activation	Increased proliferation/stemness, decreased CD8^+^ T cell infiltration	Heissenberger et al. (2019)
Pca	Tumor microenvironment	NSUN5 → PI3K-AKT signaling → macrophage polarization	Immunosuppressive TME, tumor progression	Heissenberger et al. (2019)

### 3.1 NSUN5-mediated tumor metabolism and stress adaptation

**FIGURE 2:**
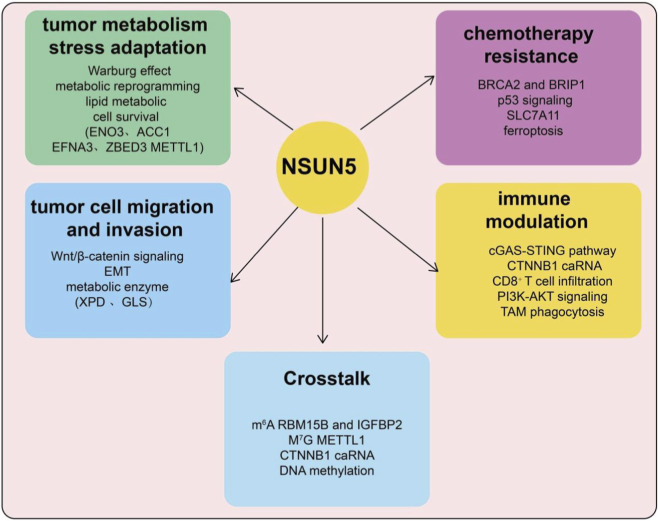
Schematic overview of NSUN5-mediated m5C modification and its roles in cancer progression and gene regulation. Conceptual model illustrating the multifaceted roles of NSUN5 in tumor biology. NSUN5-mediated RNA m^5^C modification contributes to tumor metabolic stress adaptation, including the Warburg effect, metabolic reprogramming, lipid metabolism, and cell survival pathways. NSUN5 is also implicated in tumor cell migration and invasion through regulation of Wnt/β-catenin signaling, epithelial mesenchymal transition (EMT), and metabolic enzymes. In addition, NSUN5 influences chemotherapy resistance, involving DNA damage response pathways (e.g., BRCA2/BRIP1), p53 signaling, SLC7A11 regulation, and ferroptosis. Emerging evidence suggests a role for NSUN5 in immune modulation, affecting pathways such as cGAS-STING signaling, CTNNB1-associated RNAs, CD8^+^ T-cell infiltration, PI3K-AKT signaling, and tumor-associated macrophage (TAM) phagocytosis. Crosstalk between NSUN5-dependent m^5^C modification and other epigenetic and epitranscriptomic mechanisms, including m^6^A, m^7^G, enhancer RNAs, and DNA methylation, further integrates NSUN5 into complex regulatory networks.

Accumulating evidence indicates that NSUN5 plays a critical role in tumor metabolic reprogramming, particularly by promoting glycolysis. In clear cell renal cell carcinoma (ccRCC), NSUN5 is significantly upregulated and enhances glucose uptake, lactate production, and extracellular acidification, thereby reinforcing the classic Warburg effect and supporting rapid tumor cell proliferation (Wang et al., 2023). Mechanistically, NSUN5 mediates m^5^C modification of target metabolic enzyme mRNAs, increasing their stability and protein expression, with ENO3 identified as a key downstream effector. Disruption of the NSUN5–ENO3 axis markedly reduces glycolytic activity and cell proliferation, highlighting NSUN5 as a crucial link between RNA epigenetic modification and metabolic reprogramming in cancer (Wang et al., 2023). Recent studies also show that CDK13 can phosphorylate NSUN5 at Ser327, enhancing its m^5^C methyltransferase activity. This, in turn, stabilizes ACC1 mRNA and promotes its nuclear export, facilitating fatty acid synthesis and accumulation in prostate cancer cells (Zhang et al., 2023). The CDK13–NSUN5–ACC1 axis thus reveals a novel mechanism by which NSUN5 connects RNA modification to lipid metabolic reprogramming and identifies a potential therapeutic target in prostate cancer (Zhang et al., 2023). In hepatocellular carcinoma (HCC), NSUN5 is highly expressed and stabilizes EFNA3 mRNA via m^5^C modification, promoting cell proliferation and glycolysis. EFNA3 overexpression can rescue the inhibitory effects induced by NSUN5 depletion, underscoring the functional importance of the NSUN5/EFNA3 axis in HCC progression (Han et al., 2025a). Interestingly, in gliomas, NSUN5 is silenced via DNA methylation (Figure 2). Its loss leads to demethylation at 28S rRNA C3782, globally reducing protein synthesis while activating adaptive translational programs that support cell survival under stress, suggesting a tumor-suppressive role of NSUN5 in this context, closely associated with prolonged patient survival (Yin et al., 2025).

### 3.2 Regulation of tumor cell proliferation and survival by NSUN5

NSUN5 promotes tumor cell proliferation and exerts oncogenic effects in multiple cancers (Figure 2). In HCC, elevated NSUN5 expression correlates with poor prognosis and enhances target mRNA stability via m^5^C modification, thereby activating pro-proliferative signaling pathways. Specifically, NSUN5 stabilizes ZBED3 mRNA, activating the Wnt/β-catenin signaling axis to promote tumor growth, demonstrating a critical link between RNA modification and canonical oncogenic signaling (Gu et al., 2024). In ccRCC, NSUN5 is markedly upregulated, with high expression associated with age, tumor size, TNM stage, and poor prognosis. Functional studies show that NSUN5 suppresses tumor cell senescence while promoting proliferation and migration, suggesting its potential as both a diagnostic/prognostic biomarker and a therapeutic target (Huang et al., 2024). In colorectal cancer (CRC), NSUN5 overexpression correlates with advanced tumor stage and drives cell proliferation through regulation of cell cycle proteins (e.g., CDK4, CDK6, CCNE1) and the Rb pathway (Jiang et al., 2020b). In glioblastoma (GBM), NSUN5 is overexpressed and associated with poor patient survival (Zhou et al., 2023). Mechanistically, NSUN5-mediated m^5^C modification of 28S rRNA enhances protein synthesis, promoting proliferation, sphere formation, migration, temozolomide resistance, and maintenance of tumor stemness (Zhou et al., 2023). Similarly, in esophageal and other gastrointestinal cancers, NSUN5 stabilizes METTL1 mRNA via m^5^C, accelerating proliferation and cell cycle progression (Cui et al., 2025). In cholangiocarcinoma (CCA), NSUN5 enhances GLS mRNA stability through m^5^C modification at the 137C site, promoting proliferation, migration, invasion, and resistance to cuproptosis (Shu et al., 2025). In prostate cancer (PCa), NSUN5 promotes tumor growth, migration, and immune-suppressive microenvironment formation via PI3K-AKT pathway activation and macrophage polarization (Huang et al., 2025).

### 3.3 NSUN5-driven tumor cell migration and invasion

In HCC, NSUN5 is highly expressed and recruits WDR5 to enrich H3K4me3 at the SMAD3 promoter, facilitating SMAD3-mediated epithelial–mesenchymal transition (EMT) and enhancing tumor cell migration and invasion, highlighting its role in metastasis (Han et al., 2025b). Additionally, NSUN5 (also referred to as NOP2) mediates m^5^C modification of XPD mRNA, increasing its stability and consequently suppressing proliferation, migration, and invasion, suggesting the NSUN5/NOP2–XPD axis as a potential therapeutic target. In CCA, NSUN5-mediated m^5^C modification of GLS mRNA at the 137C site stabilizes GLS protein, promoting migration, invasion, and resistance to cuproptosis, further underscoring its role in tumor progression and metabolic regulation (Sun and Ding, 2023).

### 3.4 NSUN5 in chemotherapy and radiotherapy resistance

NSUN5 overexpression contributes to chemoresistance in CRC by upregulating BRCA2 and BRIP1 and interacting with them to inhibit DNA damage-induced cell death, suggesting NSUN5 as a regulator of chemoresistance and a potential therapeutic target (Xu et al., 2025). In ccRCC, NSUN5 activates p53 signaling to suppress proliferation, migration, and invasion while promoting apoptosis, affecting drug sensitivity and highlighting its relevance as a therapeutic intervention point. NSUN5 also binds SLC7A11 mRNA, enhancing its translation and increasing resistance to ferroptosis, which is critical in acute-on-chronic liver failure (ACLF) models and regulates RNA m^5^C levels (Li et al., 2023).

### 3.5 NSUN5 in the tumor microenvironment and immune regulation

NSUN5 maintains redox homeostasis by mediating m^5^C modification of GPX4 mRNA and activates the cGAS-STING pathway, enhancing anti-tumor immune responses in colorectal adenocarcinoma (Chen et al., 2023). In gliomas, NSUN5 catalyzes m^5^C modification of CTNNB1 caRNA, recruiting TET2 to oxidize it to 5hmC. RBFOX2 recognition of 5hmC-caRNA facilitates its degradation, suppressing β-catenin expression and enhancing tumor-associated macrophage (TAM) phagocytic activity, revealing the NSUN5/TET2/RBFOX2 axis as a key mechanism linking RNA metabolism to immune regulation (Wu et al., 2024). In gastric cancer, NSUN5 overexpression correlates with tumor stage and poor prognosis, promoting proliferation, stemness, and migration via WNT/β-catenin activation while inhibiting CD8^+^ T cell infiltration, contributing to immune evasion. In PCa, NSUN5 promotes tumor progression by activating PI3K-AKT signaling and inducing pro-tumor macrophage polarization, establishing an immunosuppressive microenvironment (Liu et al., 2024).

### 3.6 Crosstalk between NSUN5-Mediated m^5^C and other epigenetic modifications

In COAD, NSUN5-mediated m^5^C of GPX4 mRNA synergizes with m^6^A modifications mediated by RBM15B and IGFBP2, collectively maintaining redox homeostasis and activating cGAS–STING signaling, highlighting functional crosstalk between RNA modifications in anti-tumor immunity (Chen et al., 2023). In esophageal cancer (ESCA), NSUN5 directly binds METTL1 transcripts and catalyzes m^5^C modification, positively regulating METTL1 expression, promoting proliferation and cell cycle progression, and reversing the tumor-suppressive effect of NSUN5 depletion (Cui et al., 2025). In gliomas, NSUN5-mediated m^5^C modification of CTNNB1 caRNA recruits TET2 for oxidation to 5hmC and RBFOX2 for degradation, downregulating β-catenin and enhancing TAM phagocytosis, demonstrating NSUN5/TET2/RBFOX2-mediated RNA crosstalk in immune microenvironment regulation (Wu et al., 2024). Furthermore, in gliomas, NSUN5 is regulated by DNA methylation; its loss shifts cytoplasmic DNA sensing from cGAS–STING-dependent to DNA-PK–HSPA8-dependent STING-independent pathways, delaying but enhancing type I interferon responses and promoting M1 polarization and chemotaxis of microglia (Wu et al., 2024). These findings reveal context-dependent tumor-suppressive activity *in vitro* yet poor clinical prognosis, highlighting the complex role of NSUN5 in tumor immune regulation.

## 4 Therapeutic strategies targeting NSUN5 and translational prospects

Therapeutic strategies targeting NSUN5 can be broadly classified into direct inhibition of its enzymatic activity and indirect modulation of related signaling pathways, while NSUN5 also holds potential as a biomarker. Direct targeting primarily relies on small-molecule inhibitors that competitively or covalently bind to NSUN5, blocking its methyltransferase activity and thereby suppressing m^5^C modification of RNA (Janin et al., 2019). The crystal structure of NSUN5 reveals potential drug-binding pockets, facilitating rational inhibitor design. However, developing specific inhibitors faces several challenges, including the highly conserved nature of the catalytic pocket, limited selectivity of potential compounds, and uncertain pharmacokinetic properties *in vivo* (Heissenberger et al., 2019). Future drug development may benefit from integrating structural biology, computational drug design, and high-throughput screening to improve both target specificity and bioactivity of candidate compounds.

Indirect targeting of NSUN5-related pathways is another feasible approach. NSUN5 modulates RNA stability and translational efficiency, thereby affecting downstream signaling axes such as mTOR and ISR, which in turn regulate tumor cell proliferation and stress adaptation. Thus, combined inhibition of these translational regulatory pathways could enhance anti-tumor efficacy (Schosserer et al., 2015). Moreover, the functional interplay between NSUN5 and other RNA modification factors (e.g., NSUN2, DNMT2) in tumors provides additional opportunities for multi-targeted intervention, enabling layered regulation of RNA epigenetic programs to improve therapeutic outcomes. Finally, NSUN5 has promising applications as a biomarker. Its expression levels and enzymatic activity can stratify patients, identifying tumor subtypes dependent on NSUN5 activity and guiding clinical treatment decisions (Tian et al., 2025). NSUN5 may also predict responses to chemotherapy, targeted therapy, or immunotherapy, facilitating personalized precision medicine. With deeper understanding of NSUN5 biology and mechanisms, its translational potential in cancer diagnosis and therapy is substantial, serving both as a therapeutic target and as a predictive biomarker for patient stratification and treatment response.

## 5 Discussion

Emerging evidence indicates that NSUN5 exhibits context-dependent functions across different cancer types. In hepatocellular carcinoma, colorectal cancer, and clear cell renal cell carcinoma, NSUN5 is frequently overexpressed, promoting tumor cell proliferation, migration, invasion, and metabolic reprogramming. Conversely, in gliomas, NSUN5 is often silenced, leading to decreased 28S rRNA m^5^C methylation and reduced global protein synthesis, which paradoxically suppresses tumor growth while activating adaptive translational programs under stress (Hu et al., 2025). The dual roles of NSUN5 may stem from tumor-type-specific differences in cellular context, metabolic demands, and microenvironmental stress, as well as the distinct repertoire of target RNAs and downstream signaling pathways. These findings suggest that NSUN5 functions as either an oncogene or tumor suppressor depending on the cellular and molecular landscape, emphasizing the importance of context-specific investigation when considering NSUN5-targeted interventions (Guarnacci et al., 2024).

The mechanisms driving NSUN5 overexpression vary among cancers. In some tumors, DNA hypomethylation at the NSUN5 promoter or enhancers may lead to transcriptional activation, whereas in others, histone modifications such as H3K4me3 or H3K27ac enrichment contribute to elevated expression. Furthermore, functional crosstalk with other RNA modification pathways, including m^6^A, may indirectly influence NSUN5 transcription or activity, forming complex epitranscriptomic regulatory networks (Guarnacci et al., 2024). These observations highlight that NSUN5 dysregulation in cancer is multifactorial, involving both epigenetic and post-transcriptional mechanisms, which may differ depending on tumor type, stage, and cellular stress conditions.

Current studies on NSUN5 in tumor immunity are still in their infancy. Preliminary data suggest that NSUN5 can modulate oxidative stress responses, macrophage polarization, and cGAS–STING signaling, yet it remains unclear whether NSUN5 directly influences immune checkpoint molecules such as PD-L1 or affects the efficacy of immunotherapies. Considering the critical role of RNA modifications in shaping the tumor immune microenvironment, further investigation is warranted to elucidate whether NSUN5 contributes to immune evasion and can be leveraged to enhance immunotherapy response. While most studies have focused on NSUN5-mediated rRNA m^5^C modification, its potential regulatory effects on noncoding RNAs (ncRNAs), including lncRNAs, circRNAs, and chromatin-associated RNAs (caRNAs), remain largely unexplored (Dominissini and Rechavi, 2017). These RNAs are known to influence transcription, epigenetic state, and RNA stability, suggesting that NSUN5 may exert broader effects on gene expression and chromatin dynamics. Investigating NSUN5-mediated m^5^C modifications on ncRNAs and caRNAs could provide novel insights into its role in tumor biology and reveal additional therapeutic opportunities (Dominissini and Rechavi, 2017). Overall, the context-specific functions, diverse regulatory mechanisms, and largely unexplored roles of NSUN5 in tumor immunity and noncoding RNA regulation underscore the complexity of its contribution to cancer biology. Addressing these gaps will not only advance our understanding of NSUN5 biology but also inform the rational design of NSUN5-targeted therapies and predictive biomarkers.

## 6 Conclusion and future perspectives

In summary, NSUN5, as an RNA m^5^C methyltransferase, plays a central role in the initiation and progression of multiple cancers. By regulating RNA stability, translation efficiency, and downstream signaling pathways, NSUN5 promotes tumor cell proliferation, migration, invasion, and stemness, while also participating in metabolic reprogramming, stress adaptation, chemoresistance, and modulation of the tumor immune microenvironment. Numerous studies indicate that NSUN5 overexpression correlates with advanced tumor stage, poor prognosis, and immune evasion, highlighting its oncogenic role and potential as both a therapeutic target and biomarker for patient stratification and treatment response. Despite these advances, current research on NSUN5 has several limitations. First, reported RNA targets and signaling pathways are not yet systematically characterized, and high-resolution, genome-wide mapping of NSUN5 targets and modification sites remains lacking. Second, most studies rely on cell lines or mouse models, with limited integration of patient-derived single-cell or spatial transcriptomic data, hindering comprehensive understanding of NSUN5 role in tumor heterogeneity and the microenvironment. Finally, while potential NSUN5 inhibitors have been proposed, systematic studies bridging mechanistic insights, drug development, and clinical translation are still limited.

Future research should focus on several directions: (i) leveraging high-throughput sequencing, single-cell, and spatial transcriptomics to accurately identify NSUN5 RNA targets and define their roles across tumor subtypes; (ii) elucidating dynamic regulatory mechanisms of NSUN5 in tumor immunity, metabolism, and stress adaptation; and (iii) integrating mechanistic studies with drug development to advance small-molecule inhibitors or combinatorial therapeutic strategies, providing viable clinical avenues for NSUN5-targeted intervention. These efforts are expected to comprehensively reveal the role of NSUN5 in tumor biology and facilitate its translational application in cancer diagnosis and therapy.

## Glossary

ccRCC: clear cell renal cell carcinoma
Pca: prostate cancer
HCC: hepatocellular carcinoma
CRC: colorectal cancer
GBM: glioblastoma multiforme
CCA: cholangiocarcinoma
GC: gastric cancer
NSUN5: NOP2/Sun RNA methyltransferase 5
m5C: 5-methylcytosine
ENO3: enolase 3
ACC1: acetyl-CoA carboxylase 1
EFNA3: ephrin-A3
ZBED3: zinc finger BED-type containing 3
GLS: glutaminase
GPX4: glutathione peroxidase 4
CTNNB1: catenin beta-1
SMAD3: SMAD family member 3
CCNE1: cyclin E1
BRCA2: breast cancer susceptibility gene 2
BRIP1: BRCA1 interacting helicase 1
WDR5: WD repeat domain 5
TET2: ten-eleven translocation methylcytosine dioxygenase 2
RBFOX2: RNA binding fox-1 homolog 2
PI3K: phosphoinositide 3-kinase
AKT: protein kinase B
Wnt: wingless-related integration site
β-catenin: beta-catenin
cGAS: cyclic GMP-AMP synthase
STING: stimulator of interferon genes
EMT: epithelial–mesenchymal transition
TAM: tumor-associated macrophage
TME: tumor microenvironment
TMZ: temozolomide
